# Hemoglobin trends and transfusion practices after cardiac arrest: a cohort study

**DOI:** 10.1186/s40560-026-00952-w

**Published:** 2026-10-15

**Authors:** Luke Andrea, Gregory Marecki, Joshua M. Kimbrell, Dennis Lin, Felice Chandra, ChihHao Yuan, Ari Moskowitz

**Affiliations:** Bronx Center for Critical Care Outcomes and Resuscitation Research, Department of Internal Medicine, Division of Critical Care Medicine, Montefiore Medical Center, Bronx, NY USA

**Keywords:** Cardiac arrest, Hemoglobin, Red blood cell transfusion, Post-cardiac arrest care

## Abstract

**Background:**

Hemoglobin targets are absent from post-cardiac arrest guidelines. New trials suggest that patients with brain and myocardial injury, injuries common post-arrest, may benefit from higher hemoglobin targets. Trends in post-arrest hemoglobin and real-world practices remain poorly characterized.

**Methods:**

We performed a retrospective cohort study of consecutive adult cardiac arrests at three academic hospitals. We screened in-hospital and out-of-hospital arrests with resuscitation efforts ongoing at hospital arrival for inclusion. Patients who survived the initial resuscitation and had a hemoglobin value measured were included. All post-arrest hemoglobin values and red blood cell transfusions were collected through 30 days; descriptive statistics and graphical visualization were used to present hemoglobin trends and transfusion practices. Exploratory analyses evaluated the association between hemoglobin and in-hospital mortality.

**Results:**

Of 2,634 cardiac arrests, 958 met inclusion criteria, with 152 (15.9%) out-of-hospital arrests and 806 (84.1%) in-hospital. The mean initial post-arrest hemoglobin was 9.75 g/dL (SD 2.60), with hemoglobin levels declining progressively over the first 20 days post-arrest. Red cell transfusions were delivered for 369 (38.5%) patients in the 30 days post-arrest, with 1,238 total transfusions. The mean pre-transfusion hemoglobin value was 6.96 g/dL (SD 1.06). Pre-transfusion hemoglobin levels were < 7 g/dL for 762 (61.6%) of transfusions, and ≥ 7 g/dL for 476 (38.4%) of transfusions. Over 30 days, the highest proportion of patients transfused was in the first 48 h. Patients with initial post-arrest hemoglobin ≥ 9 g/dL had lower in-hospital mortality (adjusted OR 0.62, 95% CI 0.46–0.84, *p*<0.01). In time-varying analysis, post-arrest hemoglobin through 30 days demonstrated an L-shaped association with mortality, with 95% of the total reduction in mortality hazard captured below 9.20 g/dL.

**Conclusions:**

Anemia is common after cardiac arrest, worsening in initial survivors. There is substantial variation in pre-transfusion hemoglobin levels, reflecting clinical equipoise. Clinical trials are needed to disentangle anemia as a driver versus a marker of outcomes, and test whether the risk is modifiable.

**Supplementary Information:**

The online version contains supplementary material available at https://doi.org/10.1186/s40560-026-00952-w.

## Background

Oxygen delivery to tissues is the product of cardiac output and the arterial oxygen content, which itself is a function of hemoglobin concentration, oxygen saturation, and to a lesser extent dissolved oxygen [1]. The American Heart Association (AHA) post-cardiac arrest guidelines provide recommendations for targeting cardiac output with specific blood pressure ranges, and optimizing oxygen saturation, but there is no specific hemoglobin threshold or transfusion strategy recommended for patients post-arrest [2]. Hemoglobin is frequently abnormal in the post-arrest period due to pre-arrest comorbidity, hemodilution from resuscitation, bleeding, and critical illness, and can be directly and rapidly modified through red blood cell transfusion [3].

Emerging evidence from two recent randomized trials has shown that a higher transfusion threshold may benefit patients with brain injury (Restrictive vs Liberal Transfusion Strategy in Patients With Acute Brain Injury; TRAIN trial [4]) and myocardial injury (Restrictive or Liberal Transfusion Strategy in Myocardial Infarction and Anemia; MINT trial [5]), two organ systems commonly injured from a cardiac arrest [6]. Observational studies in cardiac arrest populations have also associated higher hemoglobin levels at the time of return of spontaneous circulation (ROSC) with improved neurologic outcomes [7–18]. In contrast, the landmark TRICC (A Multicenter, Randomized, Controlled Clinical Trial of Transfusion Requirements in Critical Care) [19] and TRISS (Lower versus Higher Hemoglobin Threshold for Transfusion in Septic Shock) [20] trials support a restrictive transfusion strategy for general critically ill and septic patients; current international guidelines recommend a restrictive strategy that delivers red blood cell transfusion when hemoglobin levels fall below 7 g/dL [21].

Real-world transfusion practices and hemoglobin trends in the post-arrest period remain poorly characterized. To address gaps in the available observational data for post-cardiac arrest patients, we performed a retrospective study of consecutive adult patients with cardiac arrest treated at three large academic hospitals. We characterize hemoglobin trends and transfusion practices post-arrest, with intent to inform the design of future prospective studies evaluating oxygen delivery targets and transfusion strategies after cardiac arrest.

## Methods

### Study design and setting

This was a cohort study of consecutive cardiac arrests at three academic hospitals within one hospital system in the Bronx, NY. None of the participating hospitals are trauma centers, and none has a post-arrest transfusion protocol. Cardiac arrests were identified retrospectively using a quality improvement registry from February 6, 2023, to December 17, 2025. The registry is populated through a mandatory, structured electronic medical record reporting workflow in which a code narrator form is completed for each consecutive cardiac arrest event. All cardiac arrests that occur within the hospital system are entered into the code narrator, and all cases undergo manual review to confirm cardiac arrest, for highly sensitive and specific case identification within the hospital system. Patients who have an arrest outside of the hospital and regain circulation prior to hospital arrival are not documented in the code narrator form and are not captured in the study. Patients who have an arrest outside of the hospital and arrive with resuscitative efforts in progress are captured. The study was approved by the local Institutional Review Board and performed under waiver of informed consent (#2021–13077).

### Participants

We included adult patients (≥ 18 years of age) who had cardiac arrest and a matching hospital encounter in the electronic health record. Only the first cardiac arrest during a hospital admission was included. Both out-of-hospital cardiac arrest (OHCA) and in-hospital cardiac arrest (IHCA) were included. Patients were excluded if the duration from cardiac arrest to death was ≤ 1 h (immediate mortality); this exclusion served to remove patients who did not achieve ROSC, did not survive long enough for a hemoglobin value to be reliably measured, and for whom the post-arrest hemoglobin would not have plausibly served as a target for intervention or influenced outcomes. We also excluded patients without any post-arrest hemoglobin values. To ensure that any missing post-arrest hemoglobin values reflected true data unavailability rather than a data extraction error, we manually reviewed all patients with no post-arrest hemoglobin values.

### Variables and definitions

Arrests starting in the emergency department, and for those admitted to hospital care areas, were considered IHCA. Arrests beginning outside the hospital were considered OHCA. For pre-arrest hemoglobin values, the closest value drawn prior to cardiac arrest was used for patients in the hospital; we did not report pre-arrest hemoglobin values for OHCA. All post-arrest hemoglobin values from complete blood count (CBC) specimens, with the associated time they were collected, were included in the analysis. Hemoglobin values are presented as grams per deciliter (g/dL).

Red blood cell transfusion was defined as the delivery of one unit of packed red blood cells. A unit of packed red blood cells is typically 300–350 ml of volume and raises hemoglobin by 1 g/dL [22]. All consecutive transfusions after the arrest start were recorded, including the date and time they were transfused. The hemoglobin value at the time of red blood cell transfusion was defined as the most recent value drawn prior to the time of the transfusion. Transfusions were included only if a post-arrest hemoglobin measurement was available prior to the transfusion, allowing assignment of a pre-transfusion hemoglobin value; this approach was used to preferentially capture transfusion practices after the initial resuscitation period had ended. The highest sequential organ failure assessment (SOFA) score was calculated for each 24-h period after the cardiac arrest. We used the original SOFA score (not SOFA-2), and the neurologic component was omitted due to limitations in electronic health record data extraction [23–25]. Data were collected until the day of discharge, death, or 30 days post-arrest, whichever occurred first; this duration was selected to match the duration of treatment in the MINT and TRAIN trials, which chose 30 and 28 days respectively, and both suggested benefit from the intervention across that time period [4, 5]. All outcome analyses were exploratory, and used in-hospital mortality, defined as the hospital admission ending in death. In-hospital mortality was chosen as it is an objective measure that can be reliably ascertained retrospectively. Neurological and functional outcomes were not abstracted due to poor and inconsistent documentation. Patients discharged alive were not considered to have died, and follow-up ended at hospital discharge.

### Primary data analysis

The primary objectives of this study were to describe hemoglobin trends and transfusion practices after cardiac arrest. Patient characteristics were reported using counts with percentages, means with standard deviations (SD), or medians with interquartile ranges (IQR) as appropriate. Hemoglobin values and transfusions delivered were described using summary statistics and plotted visually. The number of transfusions delivered was reported by the pre-transfusion hemoglobin level and for various time points post-arrest. Select proportions were compared using the chi-square test.

Hemoglobin trends were displayed visually using both scatterplots and, locally estimated scatterplot smoothing (LOESS) plots. Plots were generated for the overall cohort, and for multiple subgroups including arrest type (OHCA vs. IHCA), outcome (hospital survival vs. death), and initial post-arrest hemoglobin (≥ 9 g/dL vs. < 9 g/dL). For transfusion-centered analyses, time was aligned to the first transfusion delivered, and patients were stratified by the hemoglobin value (≥ 7 g/dL vs. < 7 g/dL) measured immediately before and closest to the initial transfusion. For the transfusion-centered analyses, hemoglobin trajectories both before and after the initial transfusion were displayed using scatterplots, LOESS curves, and individual patient (spaghetti) plots.

International Classification of Diseases, 10th revision (ICD10) codes were extracted for the hospital admissions when the arrest occurred; ICD10 codes for major bleeding were selected based on prior literature investigating major gastrointestinal bleeding [26]. We added additional ICD10 codes for other non-gastrointestinal bleeding, and all extracted codes are displayed in Supplemental Table 1. Summary statistics were used to report presence of major bleeding for various groupings of transfusions and hemoglobins.

### Exploratory analyses

For the exposure of initial post-arrest hemoglobin level, we selected < 9 g/dL vs. ≥ 9 g/dL; this threshold was selected based on commonly used liberal transfusion thresholds in critical care trials [4, 19, 20]. Associations with in-hospital mortality were evaluated using multivariable logistic regression, adjusting for age, sex, Charlson Comorbidity Index, and arrest type (OHCA vs. IHCA). Regression outcomes were reported as adjusted odds ratios (aOR) and 95% confidence intervals (95% CI). Alternative initial post-arrest hemoglobin thresholds (7−14 g/dL), including hemoglobin as a continuous variable with restricted cubic splines, were modeled. Vasopressor-free and ventilator-free days, captured using SOFA scores, through 30 days were also compared for initial hemoglobin of < 9 g/dL vs. ≥ 9 g/dL using the Wilcoxon rank-sum test.

For the exposure of time-varying hemoglobin values over 30 days post-arrest, hemoglobin level was used as a time-updated variable in a Cox proportional hazards model. Observation times were defined by successive hemoglobin measurements, with death assigned to the final interval where a patient died. Models were adjusted for the same covariates listed above. The proportional hazards assumption was assessed using Schoenfeld residuals; a violation was identified for sex. Non-linearity was also assessed and identified; final modeling used restricted cubic splines for hemoglobin levels with three degrees of freedom. Two sensitivity analyses were performed: (1) excluding patients with major bleeding during the hospitalization and (2) incorporating time-varying daily SOFA scores into the model. To describe the non-linear association, we reported the hemoglobin level corresponding to 95% of the modeled change by rescaling the linear predictor, and fit piecewise time-varying Cox models above and below this threshold. Results were also reported as hazard ratios (HR).

All outcome analyses were considered as exploratory and hypothesis-generating only. All analyses were performed with complete case analysis; missing data is reported in Supplemental Table 2. Summary statistics and regression model comparisons were performed with Stata/SE version 19.0 (College Station, TX, StataCorp LP), and the time varying Cox proportional hazards models were performed with R version 4.5.0 (R Foundation for Statistical Computing, Vienna, Austria).

## Results

**Fig. 1:**
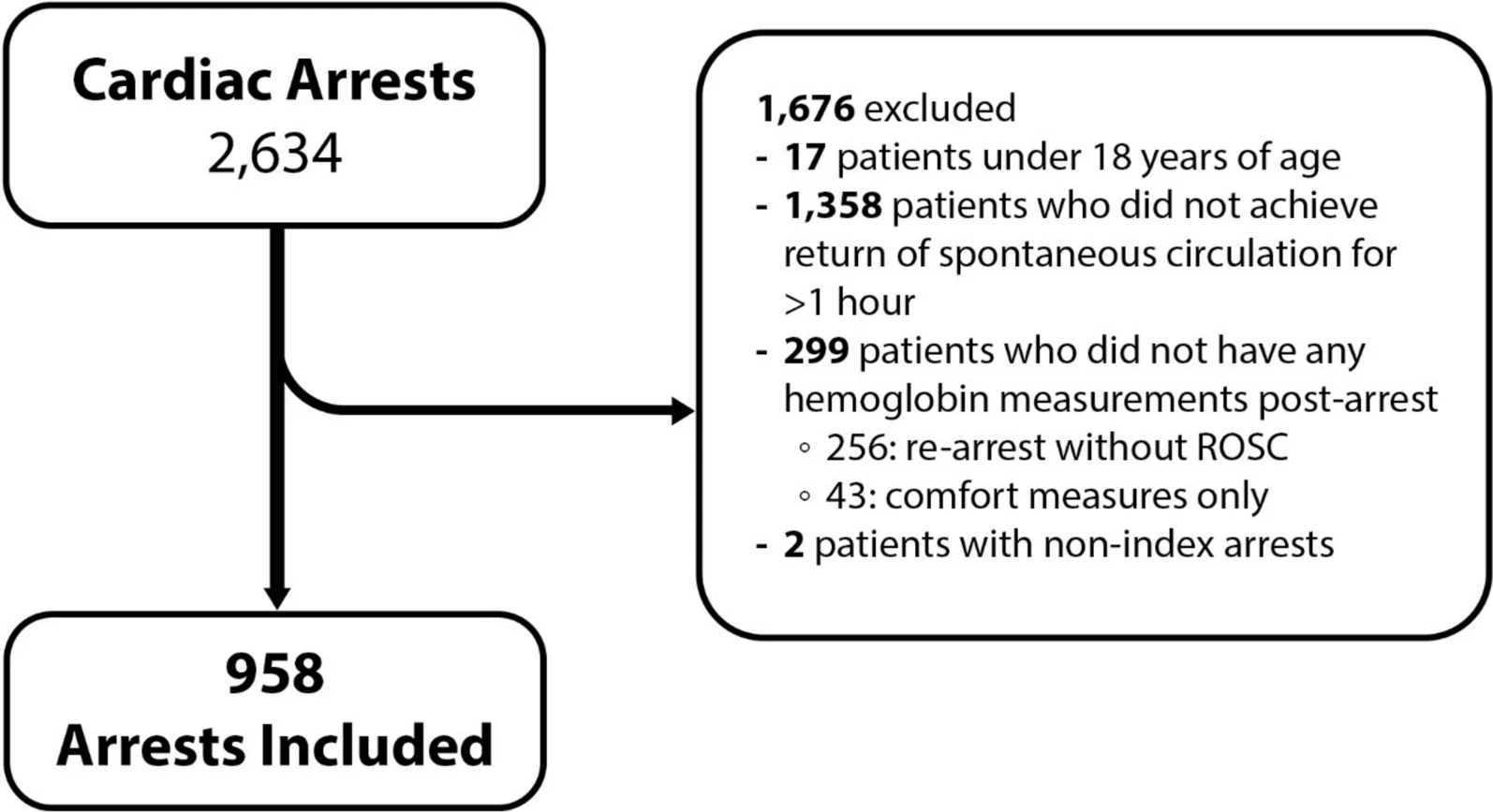
Cohort Selection Diagram

A total of 2,634 cardiac arrests were evaluated, of which 958 (36.4%) met cohort inclusion criteria (Fig. 1). Of those included, 152 (15.9%) were OHCA, 806 (84.1%) were IHCA. Patient characteristics are displayed in Supplemental Table 2. For IHCA, when a pre-arrest hemoglobin was available, the mean value was 9.51 g/dL (SD 2.18 g/dL).

### Post-arrest hemoglobin trends

For initial post-arrest hemoglobin, the mean value in was 9.75 g/dL (SD 2.60 g/dL). The median time from cardiac arrest to collection of the first hemoglobin level was 1.47 h (IQR 0.53, 5.38). Hemoglobin trends show that levels progressively decrease through the first 20 days post-arrest, reaching a nadir of 8.41 g/dL before stabilizing (Supplemental Fig. 1). Generally, for subgroups evaluated, including OHCA vs. IHCA and survivors vs. non-survivors, the hemoglobin level would decrease initially post arrest, until stabilizing at a value < 9 g/dL (Supplemental Figs. 2 through 5). When evaluated based on the initial post-arrest hemoglobin, those with initial hemoglobin < 9 g/dL generally remained < 9 g/dL (Supplemental Figs. 6 through 7). The mean hemoglobin overall within the first 24 h post-arrest was 9.69 g/dL (SD 2.42 g/dL). When stratified by arrest type, IHCA patients had lower hemoglobin than OHCA in the first 24 h after ROSC (9.49 ± 2.29 g/dL vs. 11.04 ± 2.82 g/dL).

### Post-arrest transfusion practices

**Fig. 2:**
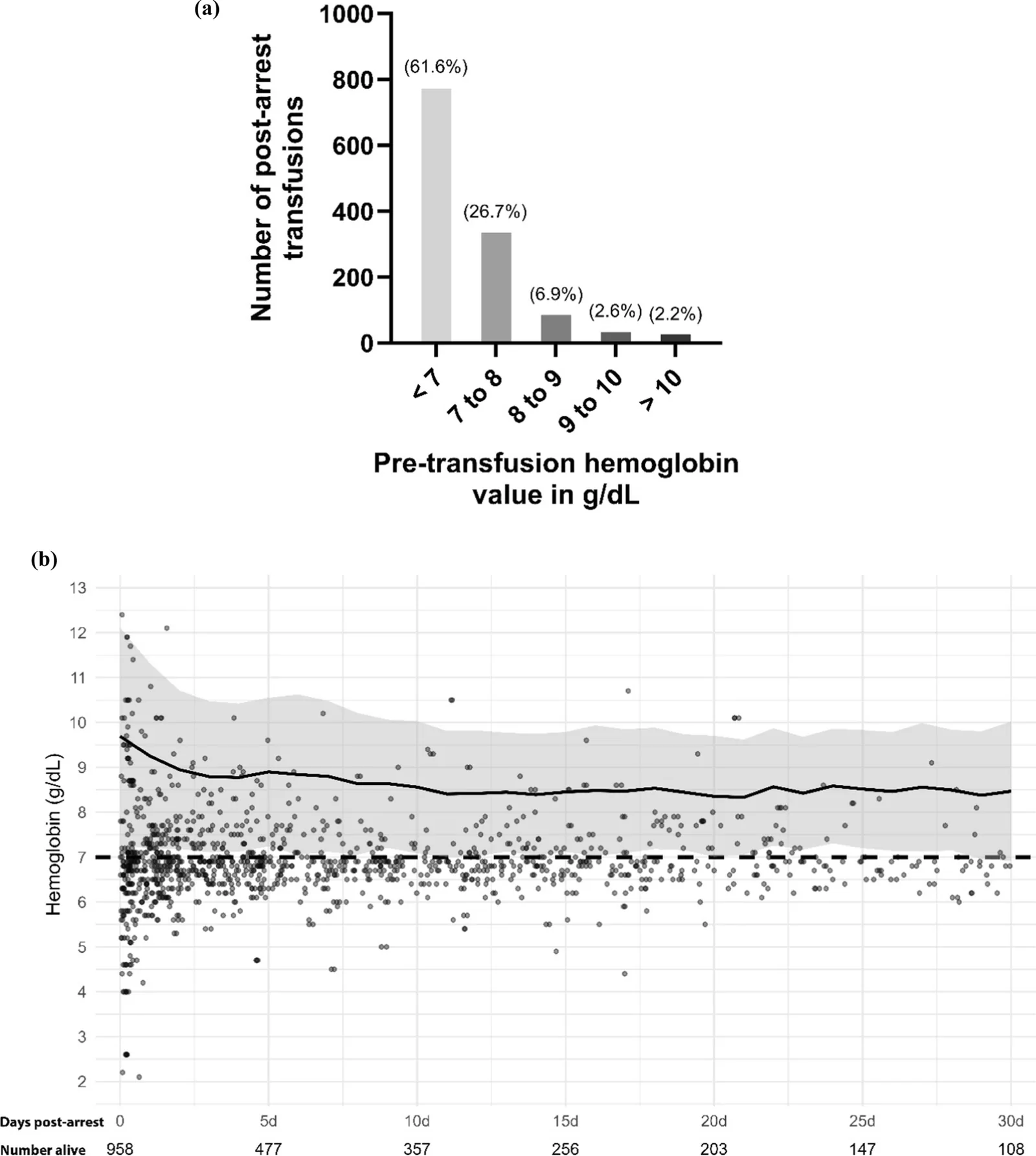
**a** Distribution of Pre-Transfusion Hemoglobin Levels. Pre-transfusion hemoglobin < 7 g/dL: median time from measurement to transfusion of 3.4 h (IQR 2.1, 5.1). Pre-transfusion hemoglobin ≥ 7 g/dL: median time from measurement to transfusion of 3.5 h (IQR 1.9, 5.8). **b** Post-Arrest Transfusions and Mean Hemoglobin. Each point represents a red blood cell transfusion plotted at the time the transfusion was delivered and at the value of the pre-transfusion hemoglobin level. The continuous line is the mean hemoglobin overall, and the shaded area is the standard deviation

There were 1,238 individual red blood cell transfusions delivered within 30 days post-arrest, with 369 (38.5%) patients receiving at least one transfusion (Supplemental Fig. 8). The mean hemoglobin value immediately pre-transfusion was 6.96 g/dL (SD 1.06 g/dL). Of all transfusions delivered, 762 (61.6%) occurred when the pre-transfusion hemoglobin value was < 7 g/dL, and 476 (38.4%) occurred when the pre-transfusion hemoglobin value was ≥ 7 g/dL (Fig. 2a). The median time from hemoglobin measurement to red blood cell transfusion was 3.4 h (IQR 2.0, 5.4); when evaluated by transfusion classification, the median and IQR was 3.4 h (2.1, 5.1) when the pre-transfusion hemoglobin was < 7 g/dL and 3.5 h (1.9, 5.8) when the pre-transfusion hemoglobin was ≥ 7 g/dL. There was variation in overall transfusion strategy per individual patient as well, with 202/369 transfused patients (54.7%) receiving ≥ 75% of their transfusions for a pre-transfusion hemoglobin value of < 7 g/dL, 72/369 (19.5%) receiving ≥ 75% of transfusions for a pre-transfusion value of ≥ 7 g/dL, and the remaining 95/369 (25.7%) receiving a mix. Patient characteristics, by transfusion classification, are displayed in Table 1. Patients with ≥ 75% transfusions with a pre-transfusion hemoglobin value of ≥ 7 g/dL had lower proportions of female sex, Black race, and had higher initial post-arrest hemoglobin values. A total of 62 patients (6.5%) had an ICD10 code for major bleeding during the hospital admission. Patients who received a red blood cell transfusion had a higher proportion of ICD10 codes for bleeding than those with no transfusion (37 [10.0%] vs. 25 [4.2%], *p*<0.01). Similarly, patients with initial post-arrest hemoglobin < 9 g/dL had a higher proportion of ICD10 codes for bleeding than those with hemoglobin ≥ 9 g/dL (33 [7.9%] vs. 29 [5.4%], *p*=0.11), although the difference was not statistically significant (Supplemental Table 3).

**Table 1:** Cohort characteristics by transfusion strategy

Variable	≥ 75% of transfusions for hemoglobin < 7 g/dL *n*=202	Mixed *n*=95	≥ 75% of transfusions for hemoglobin ≥ 7 g/dL *n*=72	Never transfused *n*=589
Age in years, mean (SD)	65.0 (14.9)	63.2 (13.4)	65.8 (11.8)	68.1 (14.3)
Female sex, *n* (%)	93 (46.0)	28 (29.5)	24 (33.3)	279 (47.4)
Race, *n* (%)				
White	19 (9.4)	7 (7.4)	9 (12.5)	87 (14.8)
Black	98 (48.5)	49 (51.6)	22 (30.6)	218 (37.0)
Other	75 (37.1)	32 (33.7)	35 (48.6)	239 (40.6)
Unknown	10 (5.0)	7 (7.4)	6 (8.3)	45 (7.6)
Arrest type, n (%)				
OHCA	21 (10.4)	12 (12.6)	7 (9.7)	112 (19.0)
IHCA	181 (89.6)	83 (87.4)	65 (90.3)	477 (81.0)
Charlson comorbidity score, mean (SD)	5.7 (3.2)	5.0 (3.0)	5.7 (3.3)	5.0 (3.0)
Days from admission to arrest for IHCA, median (IQR)	3.2 (0.1, 15.9)	2.8 (0.3, 9.1)	3.3 (0.4, 8.8)	2.8 (0.4, 10.6)
Arrest location, *n* (%)				
Intensive care unit	35 (17.3)	22 (23.2)	19 (26.4)	112 (19.0)
Emergency department	79 (39.1)	26 (27.4)	22 (30.6)	262 (44.5)
Procedural area	14 (6.9)	15 (15.8)	5 (6.9)	25 (4.2)
Hospital ward	74 (36.6)	32 (33.7)	26 (36.1)	190 (32.2)
Pre-arrest hemoglobin value in g/dL for IHCA, mean (SD)	8.64 (1.52)	9.15 (2.23)	9.57 (2.31)	9.89 (2.27)
Initial post-arrest hemoglobin				
Value in g/dL, mean (SD)	8.23 (2.03)	9.36 (2.23)	9.60 (2.04)	10.35 (2.67)

In each 48-h period post-arrest, the proportion of patients alive who received a transfusion ranged from 7.0% (576–624 h) to 21.2% (0–48 h). Transfusion rates were higher in the early post-arrest period, with the highest proportion of patients transfused occurring in the first 48 h (21.2%) and generally declining in subsequent time periods. Pre-transfusion hemoglobin values were < 7 g/dL in the majority of transfusions during nearly every 48-h period post-arrest (Table 2). There did not appear to be any clustering of transfusions for a pre-transfusion hemoglobin value ≥ 7 g/dL in the early, middle, or late post-arrest period when evaluating proportions across each 48-h interval. All red blood cell transfusions are plotted in Fig. 2b. There appears to be more variation in pre-transfusion hemoglobin values in the early post-arrest period, both when pre-transfusion hemoglobin is < 7 g/dL and ≥ 7 g/dL, with less variation occurring as time progresses post-arrest.

**Table 2:** Transfusion patterns by time interval

Hours post-arrest	Number alive and in the hospital	Number of patients who were transfused at least once (and percentage)	Number of transfusions delivered	Number of transfusions delivered for hemoglobin < 7 g/dL (and percentage)	Number of transfusions delivered for hemoglobin ≥ 7 g/dL (and percentage)
*First 48 h, in 12-h intervals*					
0–12	958	98 (10.2)	173	106 (61.3)	67 (38.7)
12–24	786	54 (6.9)	71	43 (60.6)	28 (39.4)
24–36	708	61 (8.6)	92	47 (51.6)	45 (48.9)
36–48	653	59 (9.0)	75	41 (54.7)	34 (45.3)
*Each 48-h period post-arrest*					
0–48	958	203 (21.2)	411	237 (57.7)	174 (42.3)
48–96	608	109 (17.9)	157	99 (63.1)	58 (36.9)
96–144	514	84 (16.3)	128	72 (56.2)	56 (43.8)
144–192	450	52 (11.6)	76	56 (73.7)	20 (26.0)
192–240	407	59 (14.5)	74	53 (71.6)	21 (28.4)
240–288	357	41 (11.5)	62	42 (67.7)	20 (32.3)
288–336	307	46 (15.0)	64	38 (59.4)	26 (40.6)
336–384	276	38 (13.8)	64	40 (62.5)	24 (37.5)
384–432	248	33 (13.3)	50	37 (74.0)	13 (26.0)
432–480	227	22 (9.7)	36	16 (44.4)	20 (55.6)
480–528	203	21 (10.3)	31	15 (48.4)	16 (51.6)
528–576	183	20 (10.9)	32	19 (59.4)	13 (40.6)
576–624	158	11 (7.0)	18	12 (66.7)	6 (33.3)
624–672	134	11 (8.2)	13	10 (76.9)	3 (23.1)
672–720	120	17 (14.2)	22	16 (72.7)	6 (27.3)

### Transfusion centered trends

There were 369 patients who had received at least one transfusion, included for the transfusion centered trends analysis. When centering the initial transfusion at time zero, and examining hemoglobin trajectories both before and after the transfusion (Supplemental Fig. 9a and b), we generally found acutely declining hemoglobin levels in the period preceding transfusion. For those whose pre-transfusion hemoglobin was < 7 g/dL, there was generally a pre-transfusion decline in hemoglobin, followed by an immediate increase post-transfusion. For those whose pre-transfusion hemoglobin was ≥ 7 g/dL, there was generally a gradual decline in hemoglobin both pre- and post-transfusion, with relatively little immediate change. Despite differences in immediate pre- and post-transfusion patterns, hemoglobin concentrations converged for both pre-transfusion hemoglobin < 7 g/dL and ≥ 7 g/dL approximately 1.5 days after transfusion.

### Exploratory outcomes associated with initial post-arrest hemoglobin levels

There were 417 patients (43.5%) with an initial post-arrest hemoglobin level of < 9 g/dL, and 541 (56.5%) with an initial hemoglobin level ≥ 9 g/dL. Patients with initial post-arrest hemoglobin ≥ 9 g/dL had better survival to discharge than those with < 9 g/dL (170 [31.4%] vs. 89 [21.3%]), with a lower odds of death (aOR 0.62, 95% CI 0.46–0.84, *p*<0.01) (Supplemental Table 4). Across threshold analyses (7−14 g/dL), higher initial post-arrest hemoglobin was consistently associated with a lower adjusted odds of death at all cutpoints (aOR ranging from 0.46 to 0.64), with similar discriminatory performance (Supplemental Table 5). Modeling hemoglobin continuously versus with restricted cubic splines did not demonstrate significant nonlinearity (*p*=0.56), and adjusted predicted probabilities demonstrated a graded, monotonic inverse association without any threshold effect (Supplemental Fig. 10). Alive and vasopressor-free days were heavily skewed towards zero in both groups but were greater in patients with hemoglobin ≥ 9 g/dL (median [IQR], 0 [0, 7] vs. 0 [0, 0]; p<0.01). Alive and ventilator-free days showed a similar pattern (0 [0, 8] vs. 0 [0, 0]; *p*<0.01) (Supplemental Table 6).

### Exploratory outcomes associated with hemoglobin levels through 30 days post-arrest

**Fig. 3:**
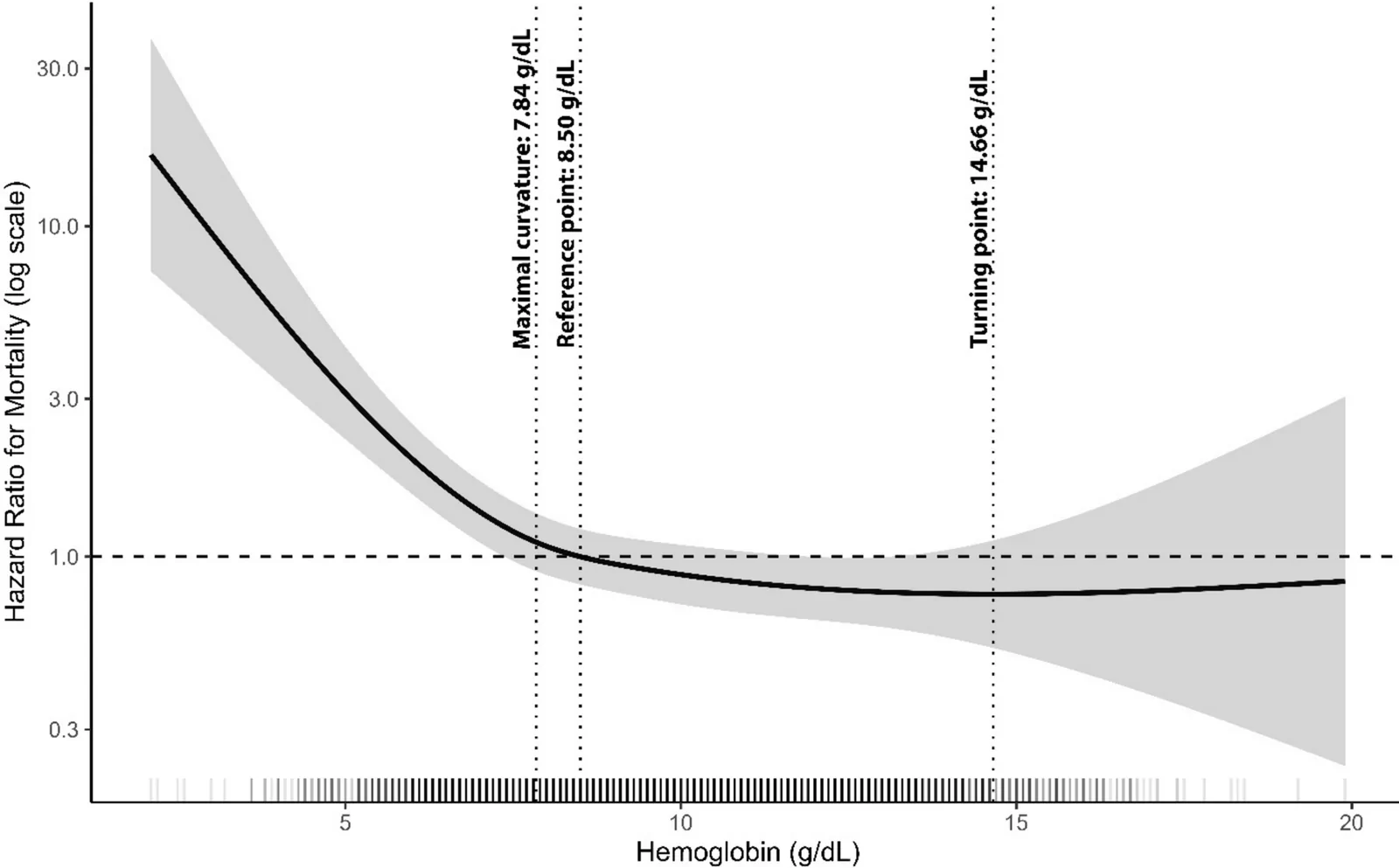
Restricted Cubic Spline Analysis of 30-Day Hemoglobin and Mortality Hazard. Hazard ratio with 95% confidence intervals (shaded region) relative to the median hemoglobin value as reference. The density of individual hemoglobin observations is displayed along the x-axis

The time-varying Cox models showed significant nonlinearity (p<0.01). Post-arrest hemoglobin demonstrated a L-shaped association with in-hospital mortality (Fig. 3). At low hemoglobin levels, there was an association between hemoglobin and the hazard of death, with 95% of the total reduction in mortality hazard captured below 9.20 g/dL, beyond which further increases in hemoglobin conferred minimal additional survival benefit. Below 9.20 g/dL, at any given time post arrest, a 1 g/dL higher hemoglobin was associated with a 29% lower instantaneous hazard of death, conditional on being alive at that time (adjusted hazard ratio of 0.71, 95% CI 0.64–0.78, p<0.01) (Supplemental Table 4). When excluding patients with an ICD10 code for major bleeding during the hospitalization, results were similar (Supplemental Fig. 11). When including the highest daily SOFA score as a covariate,t the point at which increases in hemoglobin stopped conferring any survival benefit was lower (Supplemental Fig. 12), with 95% of the total reduction in mortality hazard captured below 7.34 g/dL.

## Discussion

In this retrospective cohort study of cardiac arrest survivors, we characterize the post-arrest hemoglobin trajectory and transfusion practices in a large, contemporary cohort. Anemia post-arrest was common, with nearly half of patients having an initial value < 9 g/dL, and post-arrest trajectories showing declining hemoglobin values. Transfusions after cardiac arrest were similarly common, with transfusion demand being highest in the early post-arrest period. Although most patients received restrictive red blood cell transfusions when hemoglobin levels were < 7 g/dL, over a third received transfusions in line with a liberal transfusion strategy, with transfusions delivered when hemoglobin levels were ≥ 7 g/dL, suggesting substantial variation in post-arrest transfusion practice.

Hemoglobin is one of several determinants of oxygen delivery, alongside oxygen saturation and cardiac output [27]. However hemoglobin occupies a unique position in oxygen delivery since unlike oxygen saturation, it does not quickly reach a ceiling. Further, hemoglobin increases oxygen-carrying capacity of blood regardless of perfusion pressure, which is especially important for an injured brain with impaired autoregulation and microvascular dysfunction that is particularly dependent on arterial oxygen content rather than flow [1]. Prior post-cardiac arrest trials targeting oxygen saturations and blood pressures did not show benefit, but may have been targeting one component of the oxygen delivery equation near its physiologic maximum (oxygen saturation) and one that increases bulk flow without necessarily improving tissue-level oxygen extraction (blood pressure) [28–30].

Red blood cell transfusions delivered to increase hemoglobin levels will improve oxygen delivery, but have risks including adverse reactions and circulatory overload [22]. Landmark clinical trials have suggested that for most critically ill patients a restrictive transfusion threshold is appropriate, with the TRICC trial by Hérbert et al. finding no harm (and even potential benefit) from a restrictive transfusion strategy for patients in the intensive care unit, and the TRISS trial by Holst et al. finding no harm from a restrictive strategy in patients with septic shock [19, 20]. The findings from these landmark trials are reflected in the Association for the Advancement of Blood and Biotherapies (AABB) international red blood cell transfusion guidelines, which recommend initiating red cell transfusion when hemoglobin is < 7 g/dL (restrictive strategy) [21]. Two recent trials have explored the effect of liberal transfusion strategies on critically ill sub-populations. The MINT trial by Carson et al. found a trend towards benefit for patients with anemia and myocardial infarction treated with a liberal (≥ 10 g/dL) vs. restrictive (≥ 7 to 8 g/dL) transfusion strategy, that narrowly missed statistical significance (95% CI 0.99–1.34, *p*=0.07) [5]. The TRAIN trial by Taccone et al. found a significant benefit for patients with anemia and acute brain injury treated with a liberal (≥ 9 g/dL) vs. restrictive (≥ 7 g/dL) transfusion strategy [4]. With these new trials in mind, post-cardiac arrest patients, a subgroup characterized by brain injury and myocardial dysfunction [6], may be of potential interest for transfusion research moving forward.

The lack of long-term hemoglobin trajectory data is a critical gap in cardiac arrest research, as our data demonstrates that low hemoglobin is not confined to the early post-arrest period. Hemoglobin levels continue to decline during the weeks after ROSC, with many patients remaining in a state of poor reserve (hemoglobin < 9 g/dL) for days. This is clinically important: if the physiologic burden of anemia persists for weeks after arrest, and lower hemoglobin is associated with worse outcomes throughout that period, the window for hemoglobin optimization may extend beyond the resuscitation phase. Other studies of critically ill patients have shown that anemia is common at the time of intensive care unit admission, and that these populations experience declining hemoglobin levels over time [31–34]. Therefore, while the trends we identified in post-arrest patients may reflect critical illness rather than cardiac arrest-specific pathophysiology, characterizing these trends and distinguishing between OHCA and IHCA remains informative for post-arrest care and research.

Very few observational studies of post-arrest hemoglobin describe red blood cell transfusion practices, representing an additional gap in the literature. If transfusions are reported, studies typically only describe the proportion of patients transfused, without details on thresholds or timing [8, 11, 15]. Wormsbecker et al., in a cohort of 118 post-arrest patients, examined pre-transfusion hemoglobin values, finding inconsistent transfusion thresholds [12]. Our findings also identified variation in transfusion thresholds, with a nearly 60% to 40% split between restrictive (transfusion when hemoglobin < 7 g/dL) and liberal (transfusion when hemoglobin ≥ 7 g/dL) transfusion strategies. The variation in transfusion thresholds observed in our cohort reflects a lack of consensus on the optimal transfusion practice in post-arrest care. The observed variation does not appear to be explained by differences in clinical urgency as the time from hemoglobin measurement to transfusion was similar regardless of whether the transfusion was restrictive (when hemoglobin < 7 g/dL) or liberal (when hemoglobin ≥ 7 g/dL), and the proportion of liberal transfusions did not cluster in any particular period after the arrest, arguing against liberal transfusion (when hemoglobin ≥ 7 g/dL) being predominantly a feature of early resuscitation. Also, both liberal (when hemoglobin < 7 g/dL) and restrictive (when hemoglobin ≥ 7 g/dL) transfusions appeared to be preceded by a downtrend in hemoglobin values prior to transfusion delivery when we evaluated the first transfusion delivered post arrest. Variation was also evident at the patient level, with just over half of transfused patients receiving predominantly restrictive (when hemoglobin < 7 g/dL) transfusions and roughly one in five receiving predominantly liberal (when hemoglobin ≥ 7 g/dL) transfusions. Some differences in patient characteristics were noted between patents receiving one predominant transfusion categorization, which may reflect providers targeting different hemoglobin ranges for certain patient populations. The current AHA post-cardiac arrest guidelines provide no recommendation for transfusion strategies in this population [2]. Clinical equipoise, as demonstrated in our results, provides a natural rationale for further comparative effectiveness research into post-arrest transfusion thresholds.

As exploratory analyses, we evaluated associations between hemoglobin levels and outcomes. Multiple observational studies have associated higher hemoglobin levels near the time of arrest with better outcomes [7–18], without incorporating hemoglobin values past the first 48 h post-arrest [7, 9–18]. One exception, Albaeni et al., performed a time-varying analysis through 25 days post-arrest and found a non-significant, linear association with neurological recovery (HR 1.74, 95% CI 0.78–3.88, *p*=0.18) [8]. Our exploratory time-varying analyses through 30 days post-arrest found a non-linear association between hemoglobin and in-hospital mortality, with a plateau in effect above 9.20 g/dL (7.34 g/dL after adjusting for daily SOFA score). The larger sample size in our cohort, or the non-linear relationship we identified, may explain why we detected a significant association where Albaeni et al. did not. As with any retrospective association, these findings warrant caution.

Patients with low hemoglobin may do worse because they are more critically ill (anemia as a marker of overall illness), not because hemoglobin level modifies the post-arrest course or is itself a modifiable contributor to outcomes. Declining hemoglobin in post-arrest patients may reflect worsening critical illness through hemodilution, bone marrow suppression, or bleeding; indeed, we found a higher proportion of ICD10 codes for bleeding among patients who had received a red blood cell transfusion, although this difference in bleeding was not statistically significant between patients with a lower (< 9 g/dL) vs. higher (≥ 9 g/dL) initial post-arrest hemoglobin. Our findings were similar in a sensitivity analysis excluding patients with ICD10 codes for bleeding. The frequency of hemoglobin checks, which can also contribute to anemia, might relate to illness severity, and lower hemoglobin levels may prompt specific interventions like transfusion, which carries potential risk. Also, although we performed adjusted analyses, there were multiple potential confounders not accounted for, including baseline characteristics, arrest characteristics, and time-varying factors, as well as confounders specific to cardiac arrest including but not limited to duration of arrest, initial rhythm, and arrest etiology. Events occurring after the initial resuscitation can also act as confounders in our time-varying analyses. Most notably, withdrawal of life-sustaining therapy, where timing may be influenced by a patient’s condition or clinical trajectory, represents a particularly important unmeasured confounder in all retrospective cardiac arrest research [35]. Red blood cell transfusions, which were not accounted for in the time-varying models, may also have influenced results. For these reasons, we have explicitly stated that our outcome analyses are only exploratory. Disentangling anemia as a cause versus a consequence of deterioration is impractical in an observational framework, and clinical trials are needed.

Other limitations of this work include the capture of pre-transfusion hemoglobin levels, as the hemoglobin level immediately prior to the transfusion may not reflect the true trigger for the transfusion. Clinicians may have transfused for active bleeding, hemodynamic instability, or anticipated procedures, among other indications. Although we collected data from three hospital systems, our findings may not reflect broader transfusion practices as other institutions may practice differently, limiting generalizability. Further, our study population consisted predominantly of patients that suffered IHCA; these patients differ from those who have had OHCA in demographics, comorbidities, arrest characteristics, and outcomes, limiting the generalizability of the findings [36, 37]. This study also did not capture OHCA where ROSC was achieved in the field, prior to hospital arrival. The lack of data on the cause of cardiac arrest, including whether patients were experiencing major bleeding at the time of arrest, is another major limitation. We also do not have information on which patients were experiencing severe post-arrest brain or cardiac injury and are unable to provide analyses for these specific subgroups of interest. The lack of information regarding post-arrest neurological status means our findings may be less directly applicable to typical cardiac arrest populations in which management and prognostic strategies are focused on comatose survivors. Although we reported the outcome of survival to hospital discharge, we did not collect information on longer term outcomes or clinically important neurological or functional endpoints which are relevant for the cardiac arrest population, given that these could not be reliably obtained from simple chart review in this retrospective study. Also, survival to hospital discharge may not accurately capture more long-term survival and neurological recovery.

## Conclusions

Anemia is common after cardiac arrest and continues to worsen for most survivors of the initial resuscitation. Lower hemoglobin levels initially and throughout the weeks following a cardiac arrest are associated with worse outcomes. The substantial variation in observed transfusion practice, with clinicians employing both liberal and restrictive thresholds, reflects clinical equipoise and underscores the need for comparative effectiveness research to explore optimal hemoglobin targets after cardiac arrest.

## Supplementary Information

Additional file 1.

## Data Availability

The datasets generated and/or analyzed during the current study are not publicly available due to institutional policies, but are available from the corresponding author on reasonable request and with appropriate institutional approvals.

## Abbreviations

AHA: American heart association
TRAIN trial: Restrictive vs liberal transfusion strategy in patients with acute brain injury
MINT trial: Restrictive or liberal transfusion strategy in myocardial infarction and anemia
TRICC trial: A multicenter, randomized, controlled clinical trial of transfusion requirements in critical care
TRISS: Lower versus higher hemoglobin threshold for transfusion in septic shock
OHCA: Out-of-hospital cardiac arrest
IHCA: In-hospital cardiac arrest
ROSC: Return of spontaneous circulation
CBC: Complete blood count
SOFA: Sequential organ failure assessment
ICD10: International classification of diseases, 10th revision
SD: Standard deviation
IQR: Interquartile range
LOESS: Locally estimated scatterplot smoothing
aOR: Adjusted odds ratio
CI: Confidence interval
HR: Hazard ratio
AABB: Association for the advancement of blood and biotherapies
