# Supplementary material for: Hemoglobin trends and transfusion practices after cardiac arrest: a cohort study
Source: J Intensive Care. 2026 Oct 15;14:112. doi: 10.1186/s40560-026-00952-w (PMC13670545; doi:10.1186/s40560-026-00952-w)
Supplement: Supplementary file 1 [file 40560_2026_952_MOESM1_ESM.docx]

Contents

[Supplemental Table 1: ICD10 Codes Used	2](#_Toc237008357)

[Supplemental Table 2: Cohort Characteristics and Missingness	5](#_Toc237008358)

[Supplemental Figure 1: Hemoglobin Trajectories Overall	7](#_Toc237008359)

[Supplemental Figure 2: Hemoglobin Trajectories by Arrest Type	8](#_Toc237008360)

[Supplemental Figure 3: Hemoglobin Scatterplot by Arrest Type	9](#_Toc237008361)

[Supplemental Figure 4: Hemoglobin Trajectories by Outcome	10](#_Toc237008362)

[Supplemental Figure 5: Hemoglobin Scatterplot by Outcome	11](#_Toc237008363)

[Supplemental Figure 6: Hemoglobin Trajectory by Initial Hemoglobin	12](#_Toc237008364)

[Supplemental Figure 7: Hemoglobin Scatterplot by Initial Hemoglobin	13](#_Toc237008365)

[Supplemental Figure 8: Patient Transfusions	14](#_Toc237008366)

[Supplemental Table 3: ICD10 Codes and Transfusion Status	15](#_Toc237008367)

[Supplemental Figure 9: Hemoglobin Trajectory Centered on First Transfusion	16](#_Toc237008368)

[Supplemental Table 4: Outcomes	17](#_Toc237008369)

[Supplemental Table 5: Initial Post-ROSC Hemoglobin Outcomes	18](#_Toc237008370)

[Supplemental Figure 10: Predicted Probabilities of Death for Initial Hemoglobin	20](#_Toc237008371)

[Supplemental Table 6: Organ Failure Analyses	21](#_Toc237008372)

[Supplemental Figure 11: Outcomes Analysis Excluding Patients with Major Bleeding	22](#_Toc237008373)

[Supplemental Figure 12: Outcomes Analysis Adjusted for Time-Varying SOFA Scores	23](#_Toc237008374)

# Supplemental Table 1: ICD10 Codes Used

| **Supplemental Table 1: List of ICD10 Codes Used** | |
| --- | --- |
| **ICD10 Code** | **Name** |
| *Gastrointestinal bleeding* | |
| I85.0 | Esophageal varices with hemorrhage |
| K22.6 | Gastro-esophageal laceration-hemorrhage syndrome |
| K25.0 | Acute gastric ulcer with hemorrhage |
| K25.1 | Acute gastric ulcer with perforation |
| K25.2 | Acute gastric ulcer with both hemorrhage and perforation |
| K25.4 | Chronic or unspecified gastric ulcer with hemorrhage |
| K25.5 | Chronic or unspecified gastric ulcer with perforation |
| K25.6 | Chronic or unspecified gastric ulcer with both hemorrhage and perforation |
| K26.0 | Acute duodenal ulcer with hemorrhage |
| K26.1 | Acute duodenal ulcer with perforation |
| K26.2 | Acute duodenal ulcer with both hemorrhage and perforation |
| K26.4 | Chronic or unspecified duodenal ulcer with hemorrhage |
| K26.5 | Chronic or unspecified duodenal ulcer with perforation |
| K26.6 | Chronic or unspecified duodenal ulcer with both hemorrhage and perforation |
| K27.0 | Acute peptic ulcer, site unspecified, with hemorrhage |
| K27.1 | Acute peptic ulcer, site unspecified, with perforation |
| K27.2 | Acute peptic ulcer, site unspecified, with both hemorrhage and perforation |
| K27.4 | Chronic or unspecified peptic ulcer, site unspecified, with hemorrhage |
| K27.5 | Chronic or unspecified peptic ulcer, site unspecified, with perforation |
| K27.6 | Chronic or unspecified peptic ulcer, site unspecified, with both hemorrhage and perforation |
| K28.0 | Acute gastrojejunal ulcer with hemorrhage |
| K28.1 | Acute gastrojejunal ulcer with perforation |
| K28.2 | Acute gastrojejunal ulcer with both hemorrhage and perforation |
| K28.4 | Chronic or unspecified gastrojejunal ulcer with hemorrhage |
| K28.5 | Chronic or unspecified gastrojejunal ulcer with perforation |
| K28.6 | Chronic or unspecified gastrojejunal ulcer with both hemorrhage and perforation |
| K29.0 | Acute gastritis |
| K29.21 | Alcoholic gastritis with bleeding |
| K29.31 | Chronic superficial gastritis with bleeding |
| K29.41 | Chronic atrophic gastritis with bleeding |
| K29.51 | Unspecified chronic gastritis with bleeding |
| K29.61 | Other gastritis with bleeding |
| K29.71 | Gastritis, unspecified, with bleeding |
| K29.81 | Duodenitis with bleeding |
| K29.91 | Gastroduodenitis, unspecified, with bleeding |
| K31.82 | Dieulafoy lesion (hemorrhagic) of stomach and duodenum |
| K57.11 | Diverticulosis of small intestine without perforation or abscess with bleeding |
| K57.13 | Diverticulitis of small intestine without perforation or abscess with bleeding |
| K57.21 | Diverticulitis of large intestine with perforation and abscess with bleeding |
| K57.31 | Diverticulosis of large intestine without perforation or abscess with bleeding |
| K57.33 | Diverticulitis of large intestine without perforation or abscess with bleeding |
| K57.41 | Diverticulitis of both small and large intestine with perforation and abscess with bleeding |
| K57.51 | Diverticulosis of both small and large intestine without perforation or abscess with bleeding |
| K57.53 | Diverticulitis of both small and large intestine without perforation or abscess with bleeding |
| K57.81 | Diverticulitis of intestine, part unspecified, with perforation and abscess with bleeding |
| K57.91 | Diverticulosis of intestine, part unspecified, without perforation or abscess with bleeding |
| K57.93 | Diverticulitis of intestine, part unspecified, without perforation or abscess with bleeding |
| K62.5 | Hemorrhage of anus and rectum |
| K92.0 | Hematemesis |
| K92.1 | Melena |
| *Thoracic bleeding* | |
| I31.2 | Hemopericardium, not elsewhere classified |
| J94.2 | Hemothorax |
| R04 | Hemorrhage from respiratory passages |
| S27.1 | Traumatic hemothorax |
| *Intra-abdominal bleeding* | |
| K66.1 | Hemoperitoneum |
| K68.3 | Retroperitoneal hematoma |
| *Other bleeding* | |
| M25.0 | Hemarthrosis |
| O67 | Labor and delivery complicated by intrapartum hemorrhage, not elsewhere classified |
| O72.0 | Third-stage hemorrhage |
| O72.1 | Other immediate postpartum hemorrhage |
| O72.2 | Delayed and secondary postpartum hemorrhage |
| R31 | Hematuria |
| R58 | Hemorrhage, not elsewhere classified |

# Supplemental Table 2: Cohort Characteristics and Missingness

| **Supplemental Table 2: Cohort Characteristics and Missingness** | | | | |
| --- | --- | --- | --- | --- |
| **Variable** | **Full Cohort**  **958 patients** | **Initial Hemoglobin < 9 g/dL**  **417 patients** | **Initial Hemoglobin ≥ 9 g/dL**  **541 patients** | **Missing Proportion**  **n (%)** |
| Age in years, mean (SD) | 66.9 (14.3) | 67.5 (14.3) | 66.5 (14.2) | 0 (0) |
| Female sex, n (%) | 424 (44.3) | 195 (46.8) | 229 (42.3) | 0 (0) |
| Race, n (%) | | | | 0 (0) |
| White | 122 (12.7) | 39 (9.4) | 83 (15.3) |  |
| Black | 387 (40.4) | 195 (46.8) | 192 (35.5) |  |
| Other | 381 (39.8) | 164 (39.3) | 217 (40.1) |  |
| Unknown | 68 (7.1) | 19 (4.6) | 49 (9.1) |  |
| Arrest type, n (%) | | | | 0 (0) |
| OHCA | 152 (15.9) | 40 (9.6) | 112 (20.7) |  |
| IHCA | 806 (84.1) | 377 (90.4) | 429 (79.3) |  |
| Charlson comorbidity score, mean (SD) | 5.2 (3.0) | 5.7 (3.0) | 4.8 (3.0) | 0 (0) |
| Days from admission to arrest for IHCA, median (IQR) | 2.9 (0.3, 10.7) | 6.3 (0.9, 16.7) | 1.8 (0.1, 6.8) | 0 (0) |
| Arrest location, n (%) | | | | 0 (0) |
| Intensive care unit | 188 (19.6) | 71 (17.0) | 177 (21.6) |  |
| Emergency department | 389 (40.6) | 170 (40.8) | 219 (40.5) |  |
| Procedural area | 59 (6.2) | 31 (7.4) | 28 (5.2) |  |
| Hospital ward | 322 (33.6) | 145 (34.8) | 177 (32.7) |  |
| Pre-arrest hemoglobin value in g/dL for IHCA, mean (SD) | 9.51 (2.18) | 8.24 (1.26) | 10.85 (2.15) | 181/806 (22.5) |
| Initial post-arrest hemoglobin | | | | 0 (0) |
| Value in g/dL, mean (SD) | 9.75 (2.60) | 7.45 (1.14) | 11.51 (1.95) |  |
| Number of patients who received a red blood cell transfusion in the 30 days post-arrest, n (%) | 369 (38.5) | 220 (52.8) | 149 (27.5) | 0 (0) |

# Supplemental Figure 1: Hemoglobin Trajectories Overall

**Supplemental Figure 1: Hemoglobin Trajectories for the Total Cohort Overall After Cardiac Arrest**

Shaded areas indicate 95% confidence intervals around the LOESS-smoothed mean trajectory.

# Supplemental Figure 2: Hemoglobin Trajectories by Arrest Type

**Supplemental Figure 2: Hemoglobin Trajectories After Cardiac Arrest by Arrest Type**

Shaded areas indicate 95% confidence intervals around the LOESS-smoothed mean trajectory.

# Supplemental Figure 3: Hemoglobin Scatterplot by Arrest Type

**Supplemental Figure 3: Scatterplot of All Post-Arrest Hemoglobin Values by Arrest Type**

Shaded areas indicate 95% confidence intervals around the LOESS-smoothed mean trajectory.

# Supplemental Figure 4: Hemoglobin Trajectories by Outcome

**Supplemental Figure 4: Hemoglobin Trajectories After Cardiac Arrest by Outcome**

Shaded areas indicate 95% confidence intervals around the LOESS-smoothed mean trajectory.

# Supplemental Figure 5: Hemoglobin Scatterplot by Outcome

**Supplemental Figure 5: Scatterplot of All Post-Arrest Hemoglobin Values by Outcome**

Shaded areas indicate 95% confidence intervals around the LOESS-smoothed mean trajectory.

# Supplemental Figure 6: Hemoglobin Trajectory by Initial Hemoglobin

**Supplemental Figure 6: Hemoglobin Trajectories After Cardiac Arrest by the Initial Post-Arrest Hemoglobin**

Shaded areas indicate 95% confidence intervals around the LOESS-smoothed mean trajectory.

# Supplemental Figure 7: Hemoglobin Scatterplot by Initial Hemoglobin

**Supplemental Figure 7: Scatterplot of All Post-Arrest Hemoglobin Values by the Initial Post-Arrest Hemoglobin**

Shaded areas indicate 95% confidence intervals around the LOESS-smoothed mean trajectory.

# Supplemental Figure 8: Patient Transfusions

**Supplemental Figure 8: Distribution of Transfusions Per Patient**

# Supplemental Table 3: ICD10 Codes and Transfusion Status

| **Supplemental Table 3: Bleeding Codes by Transfusion Status** | | | | | |
| --- | --- | --- | --- | --- | --- |
| **Bleeding Type** | **Overall**  **958 patients** | **No Transfusion**  **589 patients** | **Received Transfusion**  **369 patients** | **Post-Arrest Hemoglobin < 9 g/dL**  **417 patients** | **Post-arrest Hemoglobin ≥ 9 g/dL**  **541 patients** |
| Any ICD10 code for major bleeding | 62 (6.5%) | 25 (4.2%) | 37 (10.0%) | 33 (7.9%) | 29 (5.4%) |
| ICD10 code for GI bleeding | 53 (5.5%) | 24 (4.1%) | 29 (7.9%) | 27 (6.5%) | 26 (4.8%) |
| ICD10 code for thoracic bleeding | 6 (0.6%) | 1 (0.2%) | 5 (1.4%) | 4 (1.0%) | 2 (0.4%) |
| ICD10 code for intra-abdominal bleeding | 2 (0.2%) | 1 (0.2%) | 1 (0.3%) | 2 (0.5%) | 0 (0%) |
| Other ICD10 code for bleeding | 2 (0.2%) | 0 (0%) | 2 (0.5%) | 1 (0.2%) | 1 (0.2%) |

# Supplemental Figure 9: Hemoglobin Trajectory Centered on First Transfusion

**Supplemental Figure 9a: Scatterplot With Hemoglobin Trajectory Before and After Initial Blood Transfusion After Cardiac Arrest**

Only those who received a blood transfusion (369 patients) included. Only first transfusion performed post-arrest included. Shaded areas indicate 95% confidence intervals around the LOESS-smoothed mean trajectory.

**Supplemental Figure 9b: Spaghetti Plot With Hemoglobin Trajectory Before and After Initial Blood Transfusion After Cardiac Arrest**

Only those who received a blood transfusion (369 patients) included. Only first transfusion performed post-arrest included.

# Supplemental Table 4: Outcomes

| **Supplemental Table 4: Cohort Outcomes** | | | |
| --- | --- | --- | --- |
| **Initial Post-Arrest Hemoglobin** | | | |
| **Outcome** | **Overall**  **958 patients** | **Initial Post-Arrest Hemoglobin < 9 g/dL**  **417 patients** | **Initial Post-Arrest Hemoglobin ≥ 9 g/dL**  **541 patients** |
| *Discharge disposition* | | | |
| Deceased | 699 (73.0%) | 328 (78.7%) | 371 (68.6%) |
| Home | 88 (9.2%) | 25 (6.0%) | 63 (11.7%) |
| Rehabilitation facility | 23 (2.4%) | 7 (1.7%) | 16 (3.0%) |
| Nursing facility | 121 (12.6%) | 49 (11.8%) | 72 (13.3%) |
| Hospice | 19 (2.0%) | 7 (1.7%) | 12 (2.2%) |
| Other | 8 (0.8%) | 1 (0.2%) | 7 (1.3%) |
| *Survival analyses* | | | |
| Unadjusted odds of death for initial hemoglobin ≥ 9 g/dL (compared to < 9 g/dL): 0.59  95% confidence interval: 0.44 – 0.80. p<0.01 | | | |
| Adjusted odds of death for initial hemoglobin ≥ 9 g/dL (compared to < 9 g/dL): 0.62  95% confidence interval: 0.46 – 0.84. p<0.01 | | | |
| **Time-Varying Hemoglobin Values Over 30 Days Post-Arrest** | | | |
| *Survival analysis* | | | |
| Hemoglobin < 9.20 g/dL  Adjusted hazard ratio for death per 1 g/dL hemoglobin increase: 0.71  95% confidence interval: 0.64 – 0.78. p<0.01 | | | |
| Hemoglobin ≥ 9.20 g/dL  Adjusted hazard ratio for death per 1 g/dL hemoglobin increase: 0.96  95% confidence interval: 0.87 – 1.04, p=0.31 | | | |

# Supplemental Table 5: Initial Post-ROSC Hemoglobin Outcomes

**Supplemental Table 5: Initial Post-ROSC Hemoglobin Threshold Performance**

**Initial post-ROSC hemoglobin ≥ 7 g/dL (compared to < 7 g/dL)**

Adjusted odds of death: 0.46, 95%CI 0.27 – 0.78, p<0.01

AUC = 0.60

Sensitivity: 54.4%

Specificity: 59.1%

Positive predictive value: 78.2%

Negative predictive value: 32.4%

**Initial post-ROSC hemoglobin ≥ 8 g/dL (compared to < 8 g/dL)**

Adjusted odds of death: 0.55, 95%CI 0.38 – 0.79, p<0.01

AUC = 0.60

Sensitivity: 53.2%

Specificity: 57.1%

Positive predictive value: 77.0%

Negative predictive value: 31.2%

**Initial post-ROSC hemoglobin ≥ 9 g/dL (compared to < 9 g/dL)**

Adjusted odds of death: 0.62, 95%CI 0.46 – 0.84, p<0.01

AUC = 0.60

Sensitivity: 56.4%

Specificity: 55.2%

Positive predictive value: 77.3%

Negative predictive value: 31.9%

**Initial post-ROSC hemoglobin ≥ 10 g/dL (compared to < 10 g/dL)**

Adjusted odds of death: 0.63, 95%CI 0.47 – 0.85, p<0.01

AUC = 0.60

Sensitivity: 59.4%

Specificity: 55.2%

Positive predictive value: 78.2%

Negative predictive value: 33.5%

**Initial post-ROSC hemoglobin ≥ 11 g/dL (compared to < 11 g/dL)**

Adjusted odds of death: 0.51, 95%CI 0.37 – 0.69, p<0.01

AUC = 0.62

Sensitivity: 64.2%

Specificity: 52.5%

Positive predictive value: 78.5%

Negative predictive value: 35.2%

**Initial post-ROSC hemoglobin ≥ 12 g/dL (compared to < 12 g/dL)**

Adjusted odds of death: 0.59, 95%CI 0.42 – 0.83, p<0.01

AUC = 0.60

Sensitivity: 60.7%

Specificity: 52.5%

Positive predictive value: 77.5%

Negative predictive value: 33.1%

**Initial post-ROSC hemoglobin ≥ 13 g/dL (compared to < 13 g/dL)**

Adjusted odds of death: 0.64, 95%CI 0.42 – 0.97, p=0.04

AUC = 0.60

Sensitivity: 60.2%

Specificity: 54.1%

Positive predictive value: 78.0%

Negative predictive value: 33.5%

**Initial post-ROSC hemoglobin ≥ 14 g/dL (compared to < 14 g/dL)**

Adjusted odds of death: 0.53, 95%CI 0.31 – 0.92, p=0.02

AUC = 0.59

Sensitivity: 59.7%

Specificity: 53.7%

Positive predictive value: 77.7%

Negative predictive value: 33.0%

# Supplemental Figure 10: Predicted Probabilities of Death for Initial Hemoglobin

**Supplemental Figure 10: Predicted Probabilities of In-Hospital Mortality with 95% Confidence Intervals**

Predicted probabilities of death were estimated across the range of observed initial post-arrest hemoglobin values using marginal standardization from the adjusted multivariable regression model.

# Supplemental Table 6: Organ Failure Analyses

| **Supplemental Table 6: Organ Failure Analyses** | |
| --- | --- |
| Alive and vasopressor-free days | Initial post-arrest hemoglobin ≥ 9 g/dL: median 0 days, IQR (0, 7)  Initial post-arrest hemoglobin < 9 g/dL: median 0 days, IQR (0, 0)  p<0.01 |
| Alive and ventilator-free days | Initial post-arrest hemoglobin ≥ 9 g/dL: median 0 days, IQR (0, 8)  Initial post-arrest hemoglobin < 9 g/dL: median 0 days, IQR (0, 0)  p<0.01 |

# Supplemental Figure 11: Outcomes Analysis Excluding Patients with Major Bleeding

**Supplemental Figure 11: Restricted Cubic Spline Analysis of 30-Day Hemoglobin and Mortality Hazard, Excluding Patients with Major Bleeding**

Hazard ratio with 95% confidence intervals (shaded region) relative to the median hemoglobin value as reference. The density of individual hemoglobin observations is displayed along the x-axis. There were 62 patients with an ICD10 code for major bleeding that were excluded from the analysis.

# Supplemental Figure 12: Outcomes Analysis Adjusted for Time-Varying SOFA Scores

**Supplemental Figure 12: Restricted Cubic Spline Analysis of 30-Day Hemoglobin and Mortality Hazard, Excluding Patients with Major Bleeding**

Hazard ratio with 95% confidence intervals (shaded region) relative to the median hemoglobin value as reference. The density of individual hemoglobin observations is displayed along the x-axis.
